# Case Report: A Neuro-Ophthalmological Assessment of Vision Loss in a Pediatric Case of McCune-Albright Syndrome

**DOI:** 10.3389/fmed.2022.857079

**Published:** 2022-03-15

**Authors:** Jordan D. Lemme, Anthony Tucker-Bartley, Laura A. Drubach, Nehal Shah, Laura Romo, Mariesa Cay, Stephan Voss, Neha Kwatra, Leonard B. Kaban, Adam S. Hassan, Alison M. Boyce, Jaymin Upadhyay

**Affiliations:** ^1^Department of Anesthesiology, Critical Care and Pain Medicine, Boston Children's Hospital and Harvard Medical School, Boston, MA, United States; ^2^Department of Anesthesiology, Critical Care and Pain Medicine, Massachusetts General Hospital and Harvard Medical School, Boston, MA, United States; ^3^Department of Radiology, Boston Children's Hospital, Hospital and Harvard School, Boston, MA, United States; ^4^Department of Radiology, Brigham and Women's Hospital, Hospital and Harvard School, Boston, MA, United States; ^5^Head and Neck Imaging, Department of Radiology, Massachusetts Eye and Ear and Harvard Medical School, Boston, MA, United States; ^6^Department of Oral & Maxillofacial Surgery, Massachusetts General Hospital, Harvard School of Dental Medicine, Boston, MA, United States; ^7^Eye Plastic and Facial Cosmetic Surgery, Grand Rapids, MI, United States; ^8^Metabolic Bone Disorders Unit, National Institute of Dental and Craniofacial Research, National Institutes of Health, Bethesda, MD, United States; ^9^Department of Psychiatry, McLean Hospital and Harvard Medical School, Belmont, MA, United States

**Keywords:** McCune-Albright Syndrome, craniofacial lesions, vision loss, headache, central nervous system

## Abstract

Patients diagnosed with McCune-Albright Syndrome (MAS) frequently manifest craniofacial fibrous dysplasia (FD). Craniofacial FD can impinge nerve fibers causing visual loss as well as craniofacial pain. Surgical decompression of affected nerves is performed, with variable efficacy, in an attempt to restore function or alleviate symptoms. Here, we present a case of a 12-year-old MAS patient with visual deficits, particularly in the left eye (confirmed by enlarged blind spots on Goldmann visual field testing), and craniofacial pain. Decompression surgery of the left optic nerve mildly improved vision, while persistent visual deficits were noted at a 3-month follow-up assessment. An in-depth, imaging-based evaluation of the visual system, including the retinal nerve fiber layer, optic nerves, and central nervous system (CNS) visual pathways, revealed multiple abnormalities throughout the visual processing stream. In the current FD/MAS patient, a loss of white matter fiber density within the left optic radiation and functional changes involving the left primary visual cortex were observed. Aberrant structural and functional abnormalities embedded within central visual pathways may play a role in facilitating deficits in vision in FD/MAS and contribute to the variable outcome following peripheral nerve decompression surgery.

## Introduction

Fibrous dysplasia (FD) is a rare, non-inherited bone disease arising from a R201 missense mutation of the *GNAS* gene (1–3). A key pathological feature of FD includes the formation of bony lesions within a single bone (monostotic FD) or multiple bones (polyostotic FD) (4–6). Skeletal disease may occur in isolation or in conjunction with endocrinopathies and hyperpigmented macules, termed McCune-Albright Syndrome (MAS). FD lesions readily pervade the axial-appendicular skeleton and craniofacial structures, with the latter frequently leading to craniofacial nerve compression, neuropathy, and vision loss (7–12).

Symptomatic patients with evidence of optic nerve impingement often undergo decompression surgery, yet there is variable efficacy of the procedure in terms of improving or preserving vision (13–16). Moreover, decompression surgery may cause thermal and ischemic injury to the optic nerve, resulting in neuropathy (17). We hypothesized that factors outside of isolated optic nerve impingement may facilitate visual impairment in FD/MAS patients, including those embedded within the central nervous system (CNS). To date, however, the visual processing pathways in the CNS remain unexplored in FD/MAS. We further hypothesized that in FD/MAS, the use of advanced imaging techniques that allow for characterization of CNS white matter pathways (i.e., optic radiation) and visual cortex function in parallel would provide an improved understanding of how visual deficits and, by extension, other neurological phenotypes are generated. Here, we present a pediatric MAS patient with craniofacial FD and a history of bilateral visual deficits, craniofacial pain, and headaches. In this patient, optic nerve decompression surgery in the left hemisphere improved but did not fully correct his visual deficits. To investigate the abnormalities in the visual pathway, from the retinal nerve fiber layer (RNFL) to the visual cortex, the current MAS patient was evaluated using a combination of (i) ^18^F-sodium fluoride positron emission tomography/computed tomography (^18^F-NaF PET/CT), (ii) optic coherence tomography (OCT), (iii) non-contrast, magnetic resonance imaging (MRI) of peripheral nerves, and (iv) functional and structural MRI of the CNS. This unique case and a multimodal approach provide insights into the effects of craniofacial FD on a developing biological system in addition to the complex pathophysiology of the associated neurological signs and symptoms. This report points to the importance of using a multimodal approach as early as possible upon diagnosis of pediatric FD/MAS.

## Patient Overview

A 12-year-old boy with MAS and craniofacial FD presented with worsening vision. He was diagnosed with MAS at age 8. At age 10, the patient developed visual complaints including blurry vision and decreased acuity, particularly in the left eye, which worsened over the subsequent 2 years. Functional evaluation performed with Goldmann visual field testing revealed enlarged blind spots bilaterally, with a more significant effect for the left eye than the right. Of note, he also developed bilateral chronic tension type headache pain covering regions above the eye and the posterior surface of the external ear. At age 12, visual acuity testing showed 20/70 vision and 20/100 in the right and left eye, respectively. Color testing with Ishihara was 1/11 and 0/11 in the right and left eye, respectively. OCT showed declining RNFL starting at age 10 (see Supplemental Information).

Due to concerns of declining visual acuity, the patient underwent a left orbital decompression surgery (performed by ASH) at age 12 years. The surgical procedure relieved pressure on the left optic nerve by removing a portion of the left lateral orbital wall and opening the periosteum allowing for orbital fat and the lateral rectus muscle to prolapse into the newly created space. Conservative bone removal was performed in order to reduce the likelihood of the patient developing enophthalmos. The patient had an unremarkable post-operative course. Post-operatively his vision improved to 20/40 and 20/50 in the right and left eyes, respectively, while color testing was 1/11 in both eyes. Improvements in vision were reported by the patient following surgery, but with sustained visual deficits. 3 months after the surgery, the patient presented for a multidisciplinary study of FD/MAS consisting of behavioral testing, imaging, and clinical evaluation. This study was approved by the Boston Children's Hospital and the Massachusetts General Brigham, Institutional Review Boards. The patient and patient's legal guardian provided informed consent.

### Craniofacial Pain Evaluation

At post-surgery evaluation, his craniofacial pain and headache was described as burning, shooting, stabbing, or cramping and was often triggered by bright lights, psychological stress, or physical exertion. Craniofacial pain varied between 2 and 4 on a 0–10 scale and over a one-week period. Quantitative sensory testing (QST) revealed higher cold pain threshold and tolerance temperatures in the left relative to the right craniofacial regions, specifically the V2 and V3 distribution of the trigeminal system, suggesting more cold pain sensitivity in the left hemisphere. Further details on QST procedures and findings have been provided in Supplemental Information.

### Craniofacial FD Burden

**Figure 1:**
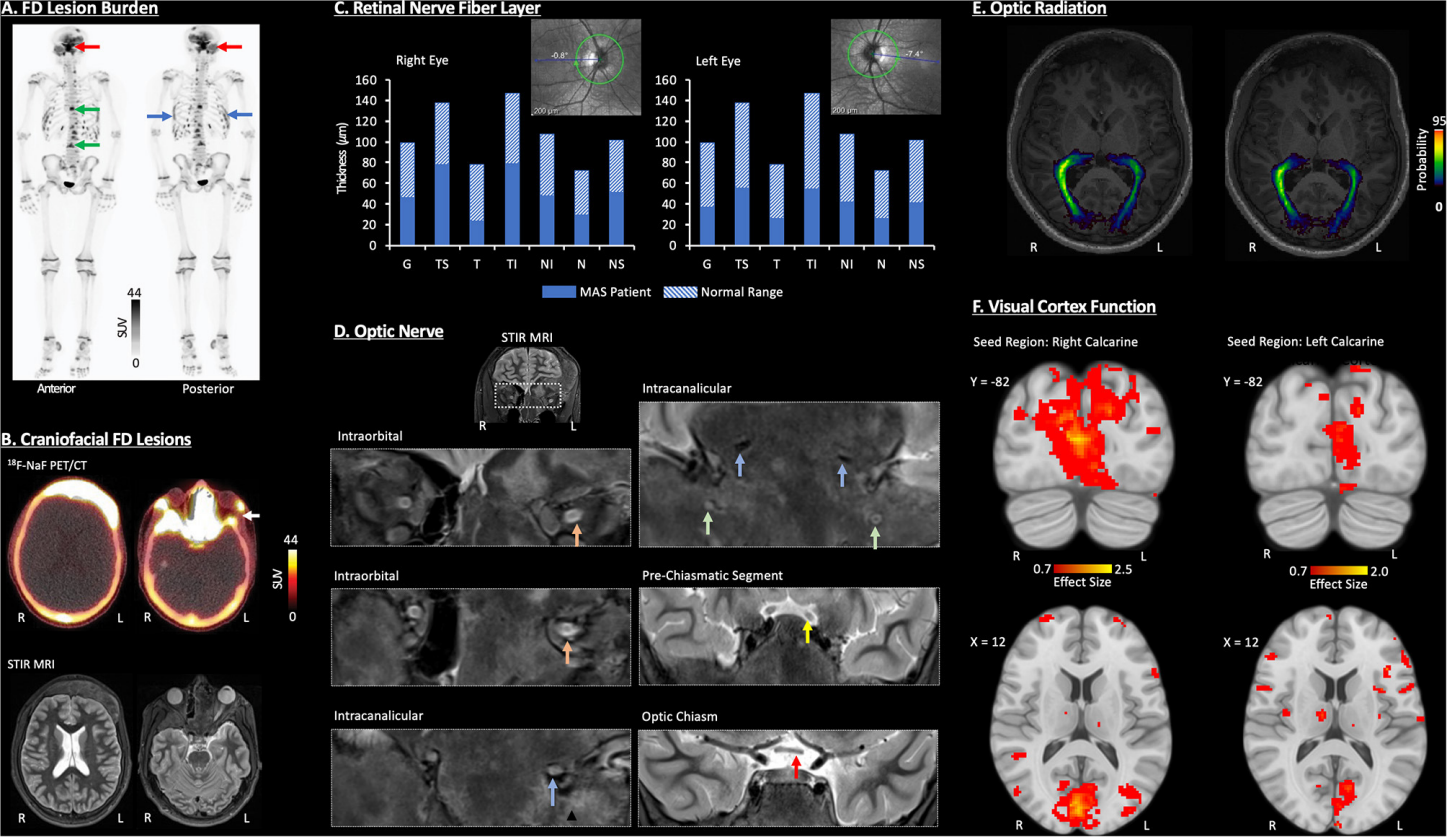
Visual pathway characterization in a pediatric MAS patient. All imaging data were acquired without sedation and at 3-months post-decompression surgery of the left optic nerve. **(A)** Whole-Body ^18^F-Sodium fluoride positron emission tomography/computerized tomography (^18^F-NaF PET/CT) identified FD lesions within craniofacial bones (red arrow), spine (green arrow), and ribs (blue arrow). **(B)** A combination of ^18^F-NaF PET/CT and non-contrast MRI identified FD in multiple craniofacial regions (i.e., frontal bones, nasal turbinates, sphenoid bone, greater wing of the sphenoid, zygomatic arch, mastoid, mandible occipital bone, and right maxillary sinus). The white arrow denotes the segment of bone tissue removed during decompression surgery of the left optic nerve. **(C)** Retinal nerve fiber layer (RNFL) loss was evident in both eyes as revealed by OCT, with slightly greater abnormalities in the left eye. G, Global Average; TS, Temporal-Superior; T, Temporal; TI, Temporal-Inferior; NI, Nasal-Inferior; N, Nasal; NS, Nasal-Superior. **(D)** Coronal, non-contrast short-tau inversion-recovery (STIR) MRI showed neuropathy in the left optic and trigeminal nerves. Inferior displacement and flattening were detected within intraorbital (orange arrow) and intracanalicular (blue arrow) segments of the left optic nerve. Bilateral trigeminal nerve (V2) inferior displacement, compression, and atrophy was also present (green arrow). At the pre-chiasmatic level (yellow arrow), inferior displacement, mild atrophy, and subtle hyperintensity indicate mild edema or gliosis of the left optic nerve. Left optic nerve atrophy was present in the optic chiasm (red arrow). **(E)** DTI revealed a loss of white matter fiber density, as defined by the probability distribution, of the left optic radiation relative to the right. The optic radiation is defined as the axonal bundle projecting between the lateral geniculate nucleus and primary visual cortex. **(F)** Resting-state functional connectivity was reduced for the left primary visual cortex compared to the right. Further details on all data acquisition and analysis procedures as well as additional study findings have been provided in Supplemental Information.

^18^F-NaF PET/CT was performed to characterize FD lesion burden and activity (Figures 1A,B). There were multiple intense foci of uptake within the skull and facial bones associated with ground-glass CT abnormalities of FD, including the frontal bone, occipital bone, right maxillary sinus, nasal turbinates, clivus, bilateral sphenoid bone, right zygomatic arch, left mastoid bone, and mandible. Furthermore, acquisition of multisequence and multiplanar, non-contrast magnetic resonance imaging (MRI) data further defined the distribution of craniofacial FD and revealed inter- and intra-lesion heterogeneity in terms of fluid content, volume, and disruption of cranial nerves (Figure 1B).

### RNFL

OCT was performed and compared to previous studies prior to surgery (Figure 1C). Following the patient's initial decline in RNFL thicknesses, at 3 months post-surgery, levels (global mean and individual quadrants) remained within the 1% thickness percentiles of the reference database. Overall, reduction in RNFL was evident in right and left eyes with, sub-regions of the temporal RNFL (i.e., Temporal-Inferior) showing more involvement in the left vs. right.

### Optic Nerve and Optic Radiation

The condition of the optic nerve and V2 division of the trigeminal system were primarily evaluated using STIR MRI (Figure 1D). At the intraorbital and intracanalicular levels, an abnormal inferior displacement of the left optic nerve was observed, and as compared to the right optic nerve, the left optic nerve was flattened, with partial effacement of the normal T2 hyperintense cerebral spinal fluid (CSF) signal within the optic nerve sheath. Intracanalicular segments of both optic nerves and V2 trigeminal nerves through foramen rotundum, showed complete effacement of the normal T2 hyperintense CSF signal along both optic nerves. There was also moderate narrowing and compression of the foramen rotundum bilaterally with atrophy of the V2 divisions of both trigeminal nerves. The pre-chiasmatic segments of both optic nerves showed a relatively smaller, inferiorly displaced left optic nerve with mild intrinsic T2 hyperintensity consistent with relative atrophy and gliosis of the left optic nerve as compared to the right. The optic chiasm showed a slightly smaller left optic nerve compared to the right consistent with left sided optic nerve atrophy.

Diffusion tensor imaging (DTI) and probabilistic tractography were performed to map the right and left optic radiation (Figure 1E). The probability density, a measure of white matter fiber density, was lower in the left optic radiation relative to the right, especially as the optic radiation nears the primary visual cortex.

### Visual Cortex Function

Functional connectivity of resting state functional MRI data was performed to evaluate primary visual cortex function (Figure 1F). In this patient, seed (right or left calcarine cortex) to voxel connectivity analysis revealed more robust connectivity for the right calcarine cortex (5,212 voxels; maximum effect size = 2.5) compared to the left calcarine cortex (1,385 voxels; maximum effect size = 2.0). The loss of functional connectivity involving the left hemisphere visual cortex reflects functional disturbance or perhaps reorganization within central visual pathways in the current MAS patient.

## Discussion

Vision loss in patients with FD/MAS is likely driven by multiple neuro-ophthalmological abnormalities and cannot be explained purely by physical optic nerve compression alone (18–20). While several case reports note improved visual function following decompression surgery (8, 9, 21), other patients with craniofacial FD fail to experience a substantial benefit (22, 23). Lastly, patients may note improved visual function without surgical decompression (24).

In many cases, the primary objective of incorporating cranial imaging in patients with FD/MAS with vison loss is to define the extent of craniofacial lesions or lesion burden, and determine whether FD structurally alters optic nerves. The impact of craniofacial FD lesions can have an affect along multiple points along the visual processing stream including those portions residing in the CNS. Here, white matter changes particularly those localized to the left optic radiation and likely maladaptive plasticity anchored within the primary visual cortex are believed to work in concert with upstream, optic nerve and RNFL abnormalities to produce the full array of visual deficits. Moreover, we hypothesize that for the current MAS patient, the impact of the craniofacial FD lesions on the left eye temporal retina as shown on OCT and downstream optic pathway (i.e., left optic radiation) alterations contributed to vision loss (see Supplemental Information). A more detailed and necessary determination of the underlying neurobiological changes causing left or right hemisphere anopia may be obtained with retinotopic mapping. A further limitation of the current study is indeed the absence of pre- *and* post-surgical DTI and fMRI datasets. With the availability of multi-timepoint neuroimaging assessments, the associations and interactions among peripheral and central visual system changes in cases of craniofacial FD could be better ascertained. The current results now provide the rationale for longitudinally incorporating DTI- and fMRI in FD/MAS studies alongside more conventional imaging methods (i.e., OCT and structural MRI of craniofacial structures) and behavioral testing of visual function. This report also details the course of a single individual with MAS; therefore, additional studies in larger and more heterogeneous populations are required to determine the generalizability of these results.

The presence of structural and functional changes between the RNFL and visual cortex may underpin vision loss either before or after decompression surgery, and relatedly the variable outcomes following surgical intervention. Moreover, while further investigation is required, the impact of craniofacial lesions on visual pathways may be unique in pediatric cases considering involvement of a developing neurobiological system. Even a mild level of FD may lead to more severe signs and symptoms, yet early and careful intervention combined with inherent neurobiological plasticity of a maturing system may facilitate better long-term outcome.

A multimodal, imaging-based assessment of the collective visual apparatus including the optic nerve, but also the orbit, RNFL, visual tracts, and the visual cortex provides an opportunity to objectively characterize the visual system and identify abnormalities that drive vision loss in FD/MAS. This complementary approach may be particularly informative when the level of patient reported visual deficit is discordant with the severity of objective FD craniofacial lesions or optic nerve compression. This strategy may be ideal to employ when there is a need to more accurately pinpoint causes of vison loss either before or after decompression surgery. Furthermore, persistent extra-skeletal abnormalities may in part explain the frequent failure of decompression surgery in alleviating visual deficits in FD; however, much work remains to thoroughly explore this perspective.

## Data Availability Statement

The original contributions presented in the study are included in the article/Supplementary Material, further inquiries can be directed to the corresponding author.

## Ethics Statement

The studies involving human participants were reviewed and approved by Boston Children's Hospital Institutional Review Board. Written informed consent to participate in this study was provided by the participant's legal guardian/next of kin.

## Author Contributions

JL, AT-B, and MC analyzed data, drafted the initial manuscript, and reviewed and revised the manuscript. LD, NS, LR, SV, and NK designed the study, acquired and analyzed data, and reviewed and revised the manuscript. LK reviewed and revised the manuscript. AH provide patient care, acquired and analyzed data, drafted the initial manuscript, and reviewed and revised the manuscript. AB designed the study, analyzed data, drafted the initial manuscript, and reviewed and revised the manuscript. JU designed the study, acquired and analyzed data, drafted the initial manuscript, and reviewed and revised the manuscript. All authors approved the final manuscript as submitted and agree to be accountable for all aspects of the work.

## Funding

This research was funded the MAYDAY Fund (PI: JU).

## Conflict of Interest

AB, through the National Institute of Dental and Craniofacial Research, receives support from Amgen, Inc for an investigator sponsored study of denosumab treatment for fibrous dysplasia. The remaining authors declare that the research was conducted in the absence of any commercial or financial relationships that could be construed as a potential conflict of interest.

## Publisher's Note

All claims expressed in this article are solely those of the authors and do not necessarily represent those of their affiliated organizations, or those of the publisher, the editors and the reviewers. Any product that may be evaluated in this article, or claim that may be made by its manufacturer, is not guaranteed or endorsed by the publisher.

## References

[1] Boyce AM Florenzano P de Castro LF Collins MT. Fibrous Dysplasia/McCune-Albright Syndrome, In: Adam MP, Ardinger HH, Pagon RA, Wallace SE, Bean LJH, Stephens K, et al. (Eds.), Seattle (WA):GeneReviews((R)) (1993).

[2] Riminucci M Liu B Corsi A Shenker A Spiegel AM Robey PG. The histopathology of fibrous dysplasia of bone in patients with activating mutations of the Gs alpha gene: site-specific patterns and recurrent histological hallmarks. J Pathol. (1999) 187:249–58. 10.1002/(SICI)1096-9896(199901)187:2<249::AID-PATH222>3.0.CO;2-J 10365102

[3] Ramaswamy G Kim H Zhang D Lounev V Wu JY Choi Y. Gsalpha controls cortical bone quality by regulating osteoclast differentiation via cAMP/PKA and beta-catenin pathways. Sci Rep. (2017) 7:45140. 10.1038/srep45140 28338087 PMC5364530

[4] Tucker-Bartley A Lemme J Gomez-Morad A Shah N Veliu MF. Upadhyay, pain phenotypes in rare musculoskeletal and neuromuscular diseases. Neurosci Biobehav Rev. (2021) 124:267Biob 10.1016/j.neubiorev.2021.02.009 PMC9521731 33581222

[5] Hartley I Zhadina M Collins MT Boyce AM. Fibrous dysplasia of bone and mccune-albright syndrome: a bench to bedside review. Calcif Tissue Int. (2019) 104:517–29. 10.1007/s00223-019-00550-z 31037426 PMC6541017

[6] Robinson C Collins MT Boyce AM. Fibrous dysplasia/mccune-albright syndrome: clinical and translational perspectives. Curr Osteoporos Rep. (2016) 14:178–86. 10.1007/s11914-016-0317-0 27492469 PMC5035212

[7] Hu AC Lee CJ Hsu FPK Vyas RM. Extensive polyostotic craniofacial fibrous dysplasia with optic nerve impingement, *J Craniofac Surg*. (2021) 32:e435–7. 10.1097/SCS.0000000000007241 33208701

[8] Bland LI Marchese MJ McDonald JV. Acute monocular blindness secondary to fibrous dysplasia of the skull: a case report. Ann Ophthalmol. (1992) 24:263–6. 1514744

[9] Papadopoulos MC Casey AT Powell M. Craniofacial fibrous dysplasia complicated by acute, reversible visual loss: report of two cases. Br J Neurosurg. 12 (1998):159–61. 10.1080/02688699845320 11013671

[10] Utriainen P Valta H Bjornsdottir S Makitie OE. Horemuzova, polyostotic fibrous dysplasia with and without mccune-albright syndrome-clinical features in a nordic pediatric cohort. Front Endocrinol (Lausanne). (2018) 9:96. 10.3389/fendo.2018.00096 29599748 PMC5863549

[11] Sarihan F Kasius KM. Teaching neuroimages: craniofacial fibrous dysplasia: loss of vision after head trauma. Neurology. (2017) 89:e236–7. 10.1212/WNL.0000000000004625 29109141

[12] Kim D Wysong A Lai J Alcorn DM Benjamin LT. Sudden onset vision loss in an 8-year-old female with McCune-Albright syndrome. Pediatr Dermatol. (2014) 31:80–2. 10.1111/j.1525-1470.2012.01800.x 23013381

[13] Seiff SR. Optic nerve decompression in fibrous dysplasia: indications, efficacy, and safety. Plast Reconstr Surg. (1997) 100:1611–2. 10.1097/00006534-199711000-00045 9385983

[14] Chen YR Breidahl A Chang CN. Optic nerve decompression in fibrous dysplasia: indications, efficacy, and safety. Plast Reconstr Surg. (1997) 99:22–30. 10.1097/00006534-199701000-00004 8982183

[15] Katz BJ Nerad JA. Ophthalmic manifestations of fibrous dysplasia: a disease of children and adults. Ophthalmology. (1998) 105:2207–15. 10.1016/S0161-6420(98)91217-9 9855148

[16] Gabbay JS Yuan JT Andrews BT Kawamoto HK Bradley JP. Fibrous dysplasia of the zygomaticomaxillary region: outcomes of surgical intervention. Plast Reconstr Surg. (2013) 13:1329–38. 10.1097/PRS.0b013e31828bd70c 23714793

[17] Shaw ML Kelley B Camarata P Sokol JA. Collateral damage: heat transfer as a possible mechanism of optic nerve injury during neurosurgical intervention. Ophthalmic Plast Reconstr Surg. (2012) 28:328–30. 10.1097/IOP.0b013e31825ca5b2 22836793

[18] Michael CB Lee AG Patrinely JR Stal S Blacklock JB. Visual loss associated with fibrous dysplasia of the anterior skull base. Case report and review of the literature. J Neurosurg. (2000) 92:350–4. 10.3171/jns.2000.92.2.0350 10659026

[19] Pan KS FitzGibbon EJ Vitale S Lee JS Collins MT Boyce SM. Utility of optical coherence tomography in the diagnosis and management of optic neuropathy in patients with fibrous dysplasia. J Bone Miner Res. (2020) 35:2199–210. 10.1002/jbmr.4129 32644197

[20] Weisman JS Hepler RS Vinters HV. Reversible visual loss caused by fibrous dysplasia. Am J Ophthalmol. (1990) 110:244–9. 10.1016/S0002-9394(14)76338-X 2396647

[21] Lin CG Wells Porrmann J Paz M Moshel YA LeBenger J Benitez RP. Organized hematoma of the sphenoid sinus with acute blindness: insight into pathogenesis of disease. Ear Nose Throat J. (2020) 99:605–9. 10.1177/0145561320941959 32692289

[22] Cutler CM Lee JS Butman JA FitzGibbon EJ Kelly MH Brillante BA. Long-term outcome of optic nerve encasement and optic nerve decompression in patients with fibrous dysplasia: risk factors for blindness and safety of observation. Neurosurgery. (2006) 59:1011–7. 10.1227/01.NEU.0000254440.02736.E3 17143235

[23] Lee JS FitzGibbon E Butman JA Dufresne CR Kushner H Wientroub S. Normal vision despite narrowing of the optic canal in fibrous dysplasia. N Engl J Med. (2002) 347:1670–6. 10.1056/NEJMoa020742 12444181

[24] Messaoud R Zaouali S Ladjimi A Ben Yahia S Jenzeri S Hmidi K. Compressive optic neuropathy caused by fibrous dysplasia. J Fr Ophtalmol. (2003) 26:631–6. 12910206

