# Supplementary material for: Case Report: A Neuro-Ophthalmological Assessment of Vision Loss in a Pediatric Case of McCune-Albright Syndrome
Source: Front Med (Lausanne). 2022 Mar 15;9:857079. doi: 10.3389/fmed.2022.857079 (PMC8964938; doi:10.3389/fmed.2022.857079)
Supplement: Supplementary file 1 [file Data_Sheet_1.pdf]

Supplemental Information

Title: A Neuro-Ophthalmological Assessment of Vision Loss in a Pediatric Case of McCune-Albright Syndrome

Jordan D. Lemme, DO[1], Anthony Tucker-Bartley, MD[1,2], Laura A. Drubach, MD[3], Nehal Shah, MD[4], Laura Romo, MD[5], Mariesa Cay, BS[1], Stephan Voss, MD[3], Neha Kwatra, MD[3], Leonard B. Kaban, MD, DMD[6], Adam S. Hassan, MD[7], Alison M. Boyce, MD[8], Jaymin Upadhyay, PhD[1,9]*

[1] Department of Anesthesiology, Critical Care and Pain Medicine, Boston Children's Hospital, Harvard Medical School, Boston, MA USA

[2] Department of Anesthesiology, Critical Care and Pain Medicine, Massachusetts General Hospital, Harvard Medical School, Boston, MA USA

[3] Department of Radiology, Boston Children's Hospital, Harvard Medical School, Boston, MA USA

[4] Department of Radiology, Brigham and Women's Hospital, Harvard Medical School, Boston, MA USA

[5] Head and Neck Imaging, Department of Radiology, Massachusetts Eye and Ear, Harvard Medical School, Boston, MA USA

[6] Department of Oral & Maxillofacial Surgery, Massachusetts General Hospital, Harvard School of Dental Medicine, Boston, MA USA

[7] Eye Plastic and Facial Cosmetic Surgery, Grand Rapids, MI USA

[8] Metabolic Bone Disorders Unit, National Institute of Dental and Craniofacial Research, National Institutes of Health, Bethesda, MD USA

[9] Department of Psychiatry, McLean Hospital, Harvard Medical School, Belmont, MA USA

Quantitative Sensory Testing (QST)

QST was performed to determine somatosensory and pain sensitivity in craniofacial areas (**Supplemental Table 1**). Pharmacological treatments to alleviate pain were not utilized by the patient. During QST, a temperature thermode (1.6x1.6 cm^2 surface area, Medoc Inc.) was placed on the subject at a baseline temperature of 32°C. The thermode was place in the V2 and V3 distribution of the trigeminal system and near the subject's upper jaw. Subject-specific warm and cool detection thresholds ('When do you first detect warmth?') were determined first, followed by heat and cold pain thresholds ('When do you first feel pain?') and cold and heat pain tolerances ('When is the stimulus too painful?') were determined. We also quantify pain thresholds corresponding to a 7/10 pain rating ('When is the pain a 7 on a 0-10 scale?'). Heat stimulation was performed prior to cold. Pressure stimulation was performed as the last QST procedure using a handheld algometer (Force Dial FDK/FDN Series Push Pull Force Gauge, Wagner Instruments). Pressure tolerance were not quantified. Each procedure was repeated three times and the average threshold or tolerance was calculated. Lower cold pain detection thresholds and cold pain tolerance temperatures were measured in the left craniofacial regions relative to the right. Otherwise, somatosensory and pain sensitivity values remained similar between right and left craniofacial areas.

QST Test	Craniofacial (Right)	Craniofacial (Left)
Warm Detection (°C)	35	36.6
Heat Pain Threshold (°C)	39.7	45
Heat Pain Tolerance (°C)	48.4	47.3
Heat 7/10 (°C)	45.8	43
Cool Detection (°C)	30.3	29
Cold Pain Threshold (°C)	7.3	18.4
Cold Pain Tolerance (°C)	1.9	5.3
Pressure Pain Detection (kgf)	1.2	1
Pressure Pain Threshold (kgf)	2.1	2.2

Supplemental Table 1. Thermal (Cold and Heat) and Pressure QST

Characterization of FD Lesion Burden with ^{18}F-NaF PET/CT

Whole-body PET/CT was performed at 3 months post-surgery (**Supplemental Fig. 1**). ^{18}F-NaF PET/CT was performed on a Siemens Biograph Vision system (Siemens, Erlangen, Germany). 4.17 mCi of ^{18}F-NaF was intravenously administrated 30 minutes prior to PET/CT acquisition. A low dose, non-diagnostic and non-contrast CT was obtained as part of the examination for purposes of anatomic localization and proton attenuation correction. There was a focus of intense ^{18}F-NaF uptake in the left frontal bone (maximum standard uptake value (SUV_{max}) = 28.6) with ground glass appearance of the skull. Additional regions of robust tracer uptake as well marked expansion of the bone included the right frontal bone, left nasal turbinates, bilateral sphenoid bone, wing of the sphenoid, and right zygomatic arch (range of SUV_{max} = 24 - 44). Other highly active lesions were observed in the clivus (SUV_{max} = 43), left mastoid (SUV_{max}= 17.2), mandible (SUV_{max}= 19), and occipital bone and right maxillary sinus that was associated with marked bone expansion (SUV_{max}= 26).

In addition to craniofacial FD lesions, the patient presented with several sites of increased uptake in the spine associated with ground glass appearance of the bone. These sites are localized at the level of C5, T1-8-9-10-11 and L1-2-3. The sites with the highest uptake are localized in T8 (SUV_{max} = 39); T11 (SUV_{max} = 30), T12 (SUV_{max} = 31.6), L1-2-3 (SUV_{max} = 38.9). Several sites of increased uptake in the sacrum also suggested additional FD lesions. Focus of higher uptake in the right anterior superior iliac spine likely represents additional FD involvement. Multiple ribs with increased uptake suggestive of FD included the right posterior second rib at the costovertebral junction, right posterior third rib, left posterior fourth rib, and multiple sites in the lower ribs bilaterally. The majority of lesions were associated with ground glass appearance and some bone expansion CT. Additional areas of uptake, for example, within articular joints, are associated with normal growth and regeneration for a 12-year old male.

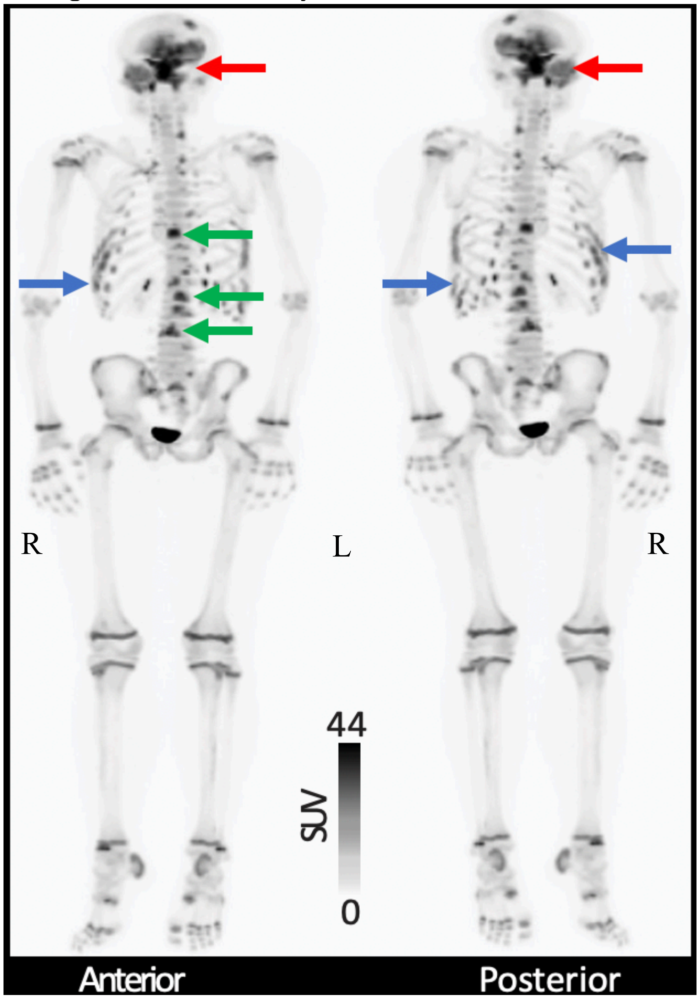

Supplemental Fig. 1. Whole-body ^{18}F-NaF PET/CT. ^{18}F-NaF PET/CT revealed a number of FD lesions in craniofacial areas (red arrows), the spine (green arrow) and ribs (blue arrow). No pain was reported outside of craniofacial anatomical sites. R = Right; L = Left; SUV: Standard Uptake Value

Characterization of FD Lesion Burden with Non-Contrast Magnetic Resonance Imaging

A multisequence and multiplanar, non-contrast magnetic resonance imaging (MRI) protocol was utilized to identify and characterized craniofacial FD. Whole head coverage was employed with exception to the implemented 3D, T2-SPACE sequence which was focused on during evaluation of cranial nerves. All MRI data were collected on a Siemens 3 Tesla Prisma scanner housed with a 64-channel head coil (Siemens, Erlangen, Germany). MRI showed normal CNS parenchyma and ventricular system. Proptosis of the left eye was evident. Overlap in craniofacial FD burden was observed between ^{18}F-NaF PET/CT and non-contrast MRI (**Supplemental Fig. 2**)

MRI Pulse sequences:
Sagittal T1. Temporal Resolution (TR) = 192 msec; Echo Time (TE) = 2.41 msec; Slice Thickness = 4 mm
Axial T1 (Thin Slice). TR = 628 msec; TE = 9.2 msec; Slice Thickness = 3.0 mm (Also acquired in the coronal plane)
Axial Short Tau Inversion Recovery (STIR). TR = 4000 msec; TE = 33 msec; Slice Thickness = 3.5 mm (Also acquired in the coronal plane)
Axial Fluid-attenuated inversion recovery (FLAIR). TR = 9000 msec; TE = 81 msec; Slice Thickness = 4.0 mm
3D T2-SPACE. TR = 1450 msec; TE = 178 msec; Slice Thickness = 600 µm
Axial Diffusion Weighted MRI (DWI). TR = 4462 msec; TE = 62 msec; Slice Thickness = 4.0 mm
Axial Proton Density + T2 Turbo Spin Echo. TR = 3600 msec; TE = 9.4 msec; Slice Thickness = 5.0 mm

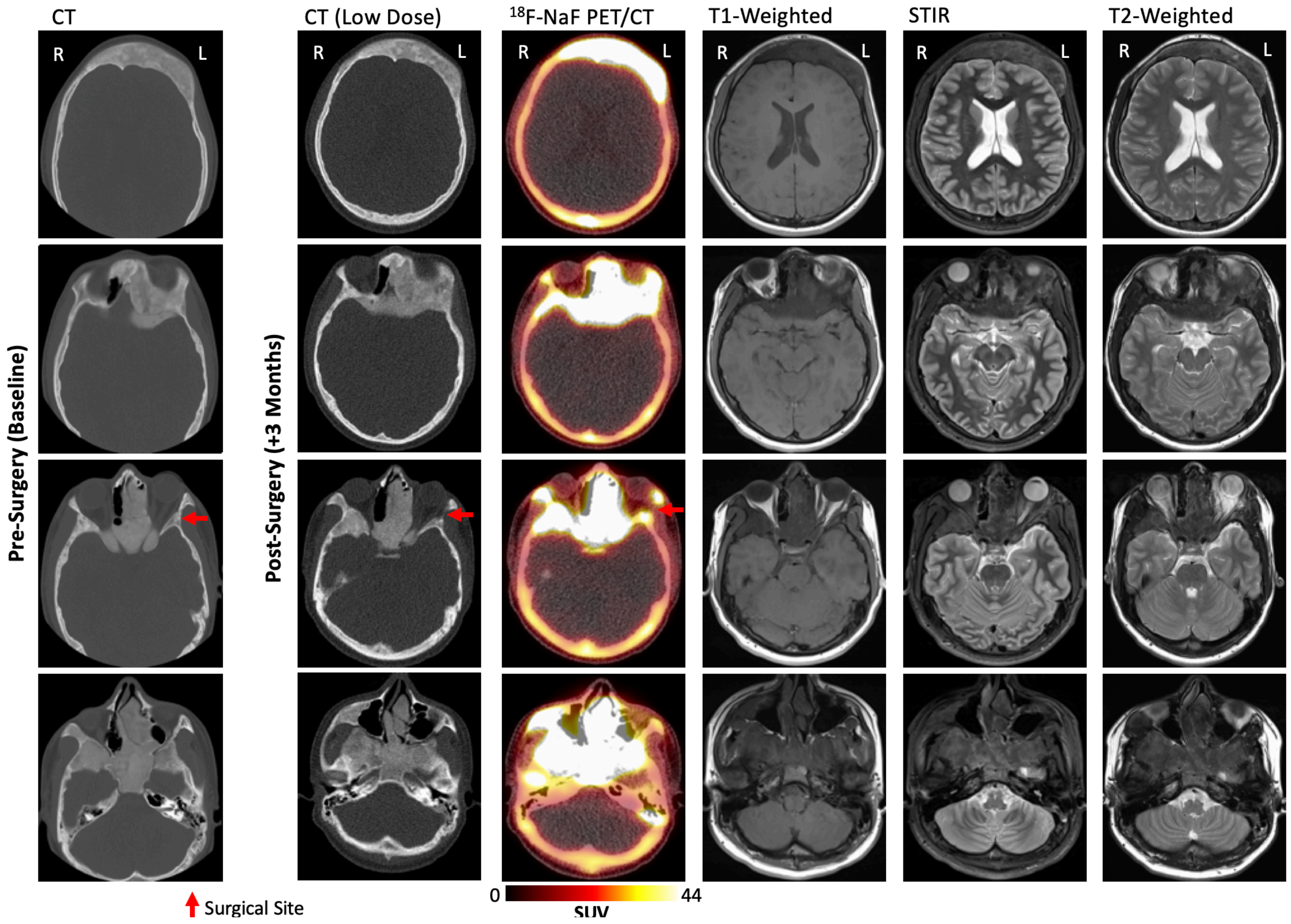

Supplemental Fig. 2. Craniofacial ^{18}F-NaF PET/CT and Non-Contrast MRI. CT was performed prior to left craniofacial surgery (red arrow). ^{18}F-NaF PET/CT and non-contrast MRI confirmed FD in a number for craniofacial bone structures as well as a greater extent of the disease in the left vs. right hemisphere. Involved bone structures include the frontal bone, nasal turbinates, sphenoid bone, wing of the sphenoid, zygomatic arch, clivus, mastoid, mandible, occipital bone, and maxilla.

Assessment of the Retinal Nerve Fiber Layer (RNFL)

Optical coherence tomography (OCT) was longitudinally performed. OCT showed declining retinal nerve fiber layers, consistent with progressive optic neuropathy. As shown in **Supplemental Fig. 3 and 4**, degradation of the RNFL was bilaterally detected 2 years prior to surgery, with progressive loss observed until the most recent evaluation time point (i.e., 3-months post-surgery). Moreover, Goldmann visual fields revealed ceco-central scotomas in each eye, the left greater than the right. The patient also had enlarged blind spots, bilaterally, which is consistent with early optic nerve damage.

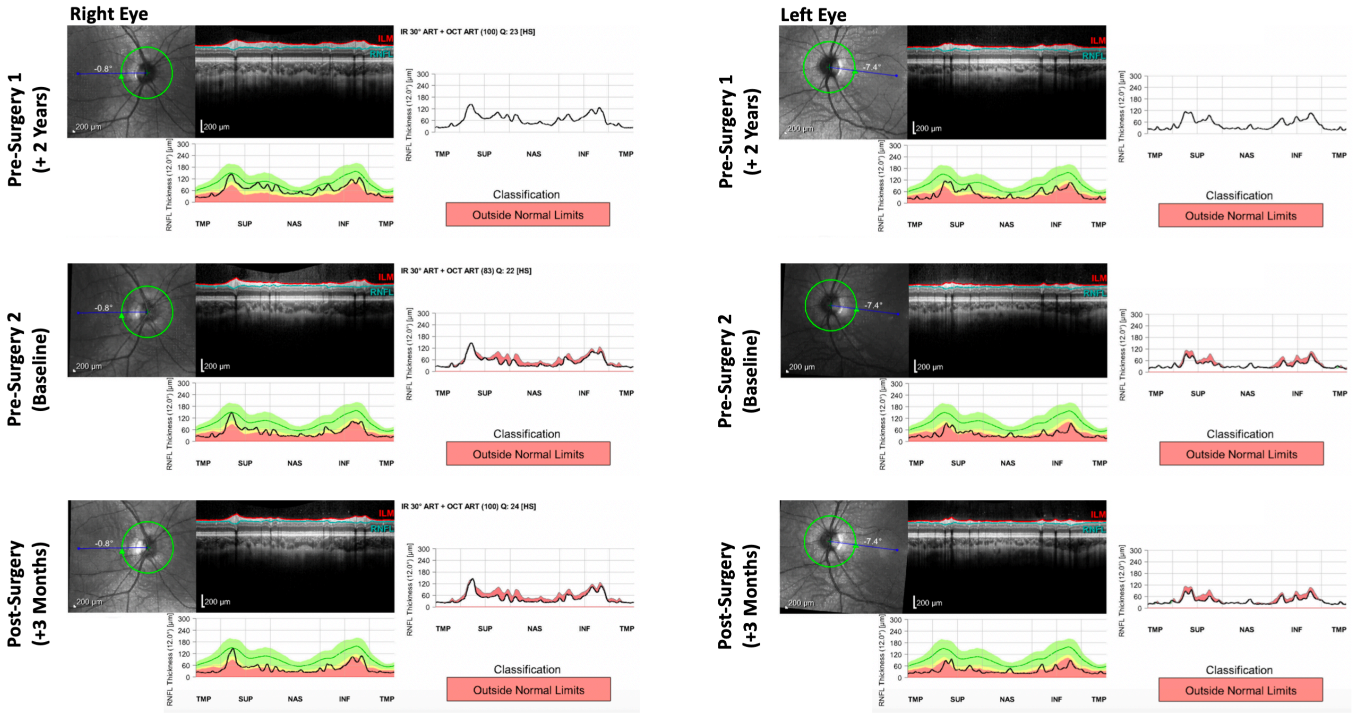

Supplemental Fig. 3. Longitudinal Assessment of RNFL with OCT. In each line graph, the color-coded indicators of green, yellow, and red correspond to 95%, 5% and 1% thickness percentiles of the reference database, respectively. TMP = temporal; SUP = superior; NAS = nasal; Inf = inferior.

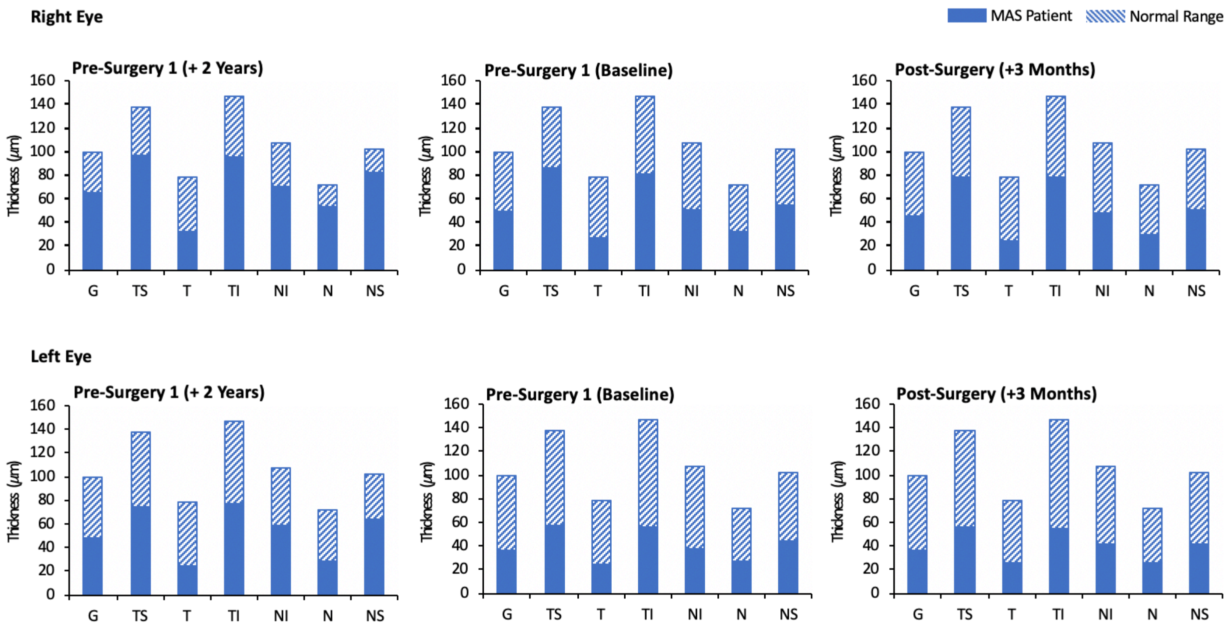

Supplemental Fig. 4. Longitudinal Assessment of RNFL with OCT (MAS vs. Normative Values). Mean values of RNFL thickness measurements (µm) provided for a global average and retinal sub-regions temporal: G = Global Average; TS = Temporal-Superior; T = Temporal; TI = Temporal-Inferior; NI = Nasal-Inferior; N = Nasal; NS = Nasal-Superior.

Structural and Functional MRI Acquisition Parameters

MRI Acquisition. Imaging protocols from The Human Connectome Project were adopted to assess CNS function and structure[1].
Structural MRI. Whole-brain, high-resolution anatomical MRI scans were acquired (multi-echo MPRAGE, 1mm^3, TR/TE1/TE2/TE3/TE4=2.53s/3.3ms/6.93ms/8.79ms/10.65ms, GRAPPA=2, 128 slices. Scan time = 4:32min.
Diffusion Tensor Imaging (DTI). DTI data were collected using the following parameters: 2.0 mm isotropic, TR/TE = 3s/64.2ms, GRAPPA=2, MB=4, b=1000s/mm^2, 80 directions, 76 slices. *Scan time = 4:54 min.*
Resting-state fMRI. During resting-state fMRI, gradient echo T2*-weighted echo-planar images were acquired using a multi-band sequence with 2 mm^3 resolution, TR/TE=800ms/37ms, 72 slices, GRAPPA=2, multiband factor=6. Scan time = 6:40min.

Structural and Functional MRI Analysis

Resting-State fMRI. A seed to voxel connectivity analysis was performed with the CONN-fMRI fc toolbox v18.b in conjunction with SPM 12 (Wellcome Department of Imaging Neuroscience, London, UK; http://www.fil.ion.ucl.ac.uk/spm/). Preprocessing included: functional realignment, slice-time corrected, spatially normalized to the Montreal Neurological Institute (MNI) space using the normalized EPI template image in SPM, and spatially smoothed with a 5-mm full-width half-maximum Gaussian kernel. Motion parameters from realignment were evaluated, and a motion artefact threshold (translation > 0.9 mm, rotation >1°) was employed for exclusion. Acquisitions with framewise displacement above 0.9mm or global BOLD signal changes above 5 s.d. are flagged as potential outliers and were excluded from analysis. BOLD data were bandpass filtered (0.008–0.09 Hz) to reduce low-frequency drift and noise effects. Individual correlation maps were generated in the CONN toolbox by extracting the mean resting-state BOLD time course from each seed ROI and calculating correlation coefficients with the BOLD time course of each voxel throughout the whole brain.

DTI. Cortical reconstruction and volumetric segmentation were performed with Freesurfer (version 7.2) image analysis suite[2-7]. Once the cortical models were completed, parcellation of the cerebral cortex into regional units were completed[5, 8]. This method produced representations of cortical thickness and sub-cortical volume[5]. For data analyzed in this study, all surfaces were visually checked, and manual interventions were used as needed to correct small defects. Next, the diffusion-weighted images, underwent trac-all in Freesurfer correcting for image distortions and B0 inhomogeneities. Registration of head motion and the diffusion-weighted image to the anatomical image. Pathway reconstruction was lastly completed[9].

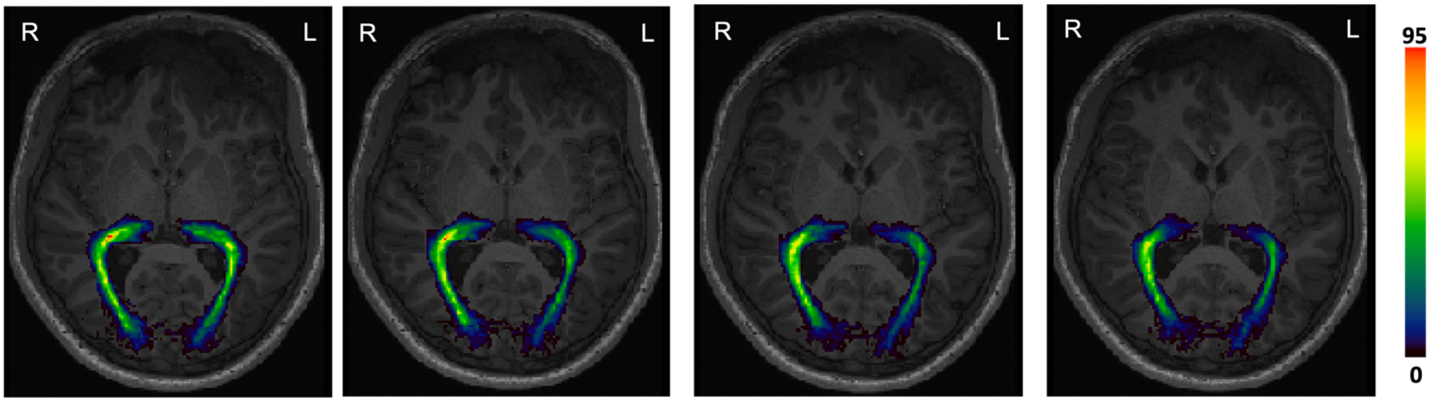

Supplemental Fig. 5. Diffusion Tensor Imaging. Probabilistic Tractography of the Right (R) and Left (L) Optic Radiation (OR) Post-Surgery (+3 Months). Left & Right OR Volumes = 2408 and 2445 voxels, respectively. Fractional Anisotropy averaged over the entire Left OR = 0.499 and entire Right OR = 0.515.

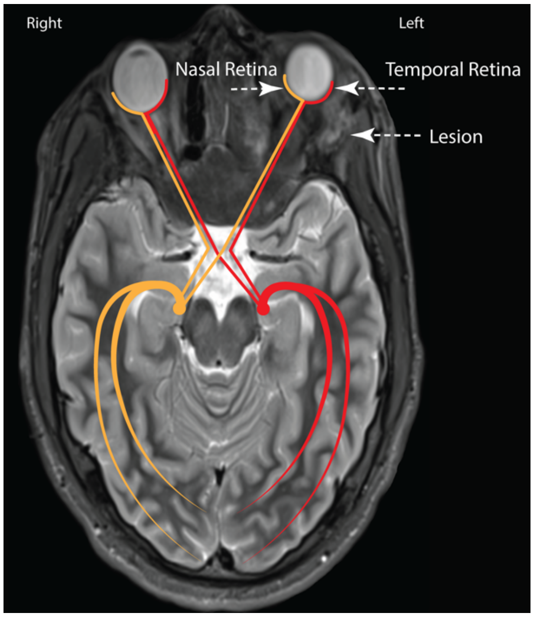

Supplemental Fig. 6. Optic pathway projections. Optic pathways spanninig between the eye and visual cortex are shown on a STIR MRI axial slice. The presense of craniofacial FD lesions proximal to the left temporal retina may explain in part the deficits in the left visual field experienced by the MAS patient and also, downstream alterations within the left sided visual system.

References

1. Glasser MF, Smith SM, Marcus DS, Andersson JL, Auerbach EJ, Behrens TE, et al. The Human Connectome Project's neuroimaging approach. *Nat Neurosci*. 2016;19(9):1175-1187.
2. Dale AM, Sereno MI. Improved Localizadon of Cortical Activity by Combining EEG and MEG with MRI Cortical Surface Reconstruction: A Linear Approach. *J Cogn Neurosci*. 1993;5(2):162-176.
3. Sled JG, Zijdenbos AP, Evans AC. A nonparametric method for automatic correction of intensity nonuniformity in MRI data. *IEEE Trans Med Imaging*. 1998;17(1):87-97.
4. Dale AM, Fischl B, Sereno MI. Cortical surface-based analysis. I. Segmentation and surface reconstruction. *Neuroimage*. 1999;9(2):179-194.
5. Fischl B, Dale AM. Measuring the thickness of the human cerebral cortex from magnetic resonance images. *Proc Natl Acad Sci U S A*. 2000;97(20):11050-11055.
6. Fischl B, Liu A, Dale AM. Automated manifold surgery: constructing geometrically accurate and topologically correct models of the human cerebral cortex. *IEEE Trans Med Imaging*. 2001;20(1):70-80.
7. Fischl B, Salat DH, Busa E, Albert M, Dieterich M, Haselgrove C, et al. Whole brain segmentation: automated labeling of neuroanatomical structures in the human brain. *Neuron*. 2002;33(3):341-355.
8. Desikan R, Lee I, Thundat T. Effect of nanometer surface morphology on surface stress and adsorption kinetics of alkanethiol self-assembled monolayers. *Ultramicroscopy*. 2006;106(8-9):795-799.
9. Yendiki A, Panneck P, Srinivasan P, Stevens A, Zollei L, Augustinack J, et al. Automated probabilistic reconstruction of white-matter pathways in health and disease using an atlas of the underlying anatomy. *Front Neuroinform*. 2011;5:23.
